# Thickness change of masseter muscles and the surrounding soft tissues in female patients during orthodontic treatment: a retrospective study

**DOI:** 10.1186/s12903-020-01168-6

**Published:** 2020-06-29

**Authors:** Yichen Pan, Si Chen, Linhui Shen, Yuru Pei, Yungeng Zhang, Tianmin Xu

**Affiliations:** 1 Department of Orthodontics, Peking University School and Hospital of Stomatology, 22 Zhongguancun Avenue South, Haidian District, Beijing, 100081 PR China; 2 National Engineering Laboratory for Digital and Material Technology of Stomatology, 22 Zhongguancun Avenue South, Haidian District, Beijing, 100081 PR China; 3 Beijing Key Laboratory of Digital Stomatology, 22 Zhongguancun Avenue South, Haidian District, Beijing, 100081 PR China; 4 Third Clinical Division, Peking University School and Hospital of Stomatology, Block A, Gaode Building, Huayuan East Road, Haidian District, Beijing, 100083 PR China; 5 Key Laboratory of Machine Perception (MOE), Department of Machine Intelligence, Peking University, Science Building 2, No.5 Yiheyuan Road Haidian District, Beijing, 100871 PR China

**Keywords:** Masseter muscles, CBCT, Machine learning, Computer-assisted image processing

## Abstract

**Background:**

Facial esthetics is a major concern of orthodontic patients. This study aims to evaluate orthodontic treatment-related thickness changes of the masseter muscles and surrounding soft tissues and the potential factors that would influence these changes during orthodontic treatment in female adults.

**Methods:**

Forty-two female adult patients were included in this retrospective study and were divided into extraction (*n* = 22) and nonextraction (*n* = 20) groups. Pretreatment and posttreatment cone-beam computed tomography (CBCT) images were superimposed and reconstructed. The thickness changes of the masseter area of facial soft tissue (MAS), masseter muscles (MM) and surrounding fat tissue (FT) were measured. Pretreatment age, treatment duration, sagittal relationship (ANB), and vertical relationship (Frankfort-mandibular plane angle, FMA)-related MAS, MM and FT changes were compared between extraction and nonextraction groups. Spearman’s correlation coefficient was calculated between the above variables. Regression analysis was conducted to confirm the causal relations of the variables.

**Results:**

The thickness of MAS and MM significantly decreased in both groups, with larger decreases (> 1 mm) in the extraction group. There were strong correlations (*r* > 0.7) between the thickness decrease in MAS and MM in both groups and moderate correlations (*r* > 0.4) between MAS and FT in the nonextraction group. A significantly greater decrease of MAS and MM were found to be moderately correlated with a smaller FMA (*r* > 0.4) in the extraction group. Scatter plots and regression analysis confirmed these correlations.

**Conclusions:**

Masseter muscles and the surrounding soft tissue exhibited a significant decrease in thickness during orthodontic treatment in female adults. Low-angle patients experienced a greater decrease in soft tissue thickness in the masseter area in the extraction case. But the thickness changes were clinically very small in most patients.

## Background

Changes in the morphology of facial soft tissues have been a major concern of orthodontic patients, especially for female adults. Previous studies mostly focused on the change in lips or chins after orthodontic treatment since they were more directly related to the teeth position change [1–4]. However, noticeable changes also occur in the frontal view, sometimes in an unaesthetic way, especially in the adult female group. Some patients complained about the concave contour in the buccal area after treatment, which was viewed as a sign for aging in their opinions [5]. Did these changes result from teeth movement? To what extent orthodontic teeth movement might influence the soft tissues in the buccal area? Some studies were conducted to explore the answer. At first, digital photographs in the frontal view were used [6, 7]. Bishara et al. [6] used the distance between the left and right soft tissue gonion point from the frontal digital photographs to study the change of facial width during the growth of adolescents. The development of 3D imaging systems [8] made it possible to display the facial morphology in three dimensions. Many studies analyzed the overall facial morphological changes during orthodontic treatment using 3D surface scanning [9–11]. Moss et al. [10] found decreases in facial width in both extraction and nonextraction groups in adolescents. Ismail et al. [9] described flatter cheeks after treatment in the extraction group in adolescents. Furthermore, Dai et al. [5] analyzed the morphological change of specifically the buccal area in female adult patients and found that in extraction cases, the depth of the facial buccal area significantly decreased during orthodontic treatment while no significant change was found in the nonextraction group.

From the frontal view, the soft tissue gonion area, which is mainly supported by the masseter muscle, has a great influence on facial width. From the previous studies, changes in the underlying masseter muscle morphology could be a factor causing facial morphological changes in the buccal area [12, 13]. However, to our knowledge, there has been no study exploring the change of the masseter muscle and its relationship with the morphological change of the surrounding soft tissue in adult patients who accepted only orthodontic treatment. Previous studies showed that muscle tissue could be distinguished [14] and measured [15] using cone-beam computer tomography (CBCT). In the meantime, the facial surface can also be segmented from CBCT for 3D reconstruction. Therefore, in this study, pre- and posttreatment CBCT data were collected with the aim of evaluating the orthodontic treatment-related thickness and morphological changes of the masseter muscles and surrounding soft tissues as well as the potential factors that would influence these changes during orthodontic treatment in female adults. The null hypothesis was that there was no significant change in masseter muscles and surrounding soft tissues after extraction and nonextraction orthodontic treatments in adult female patients.

## Methods

### Patients and CBCT scans

This study was a retrospective study. Pre- and posttreatment CBCT images of 42 female patients were retrieved from the archives of National Engineering Laboratory for Digital and Material Technology of Stomatology and Beijing Key Laboratory of Digital Stomatology originally collected from 2009 to 2019. The patients all accepted orthodontic treatment in the Department of Orthodontics, Peking University School and Hospital of Stomatology. The inclusion and exclusion criteria were: (1) female patients between 18 to 35 years old whose body weight change during the treatment period was less than 2 kg, (2) CBCT images which included the whole masseter muscle and corresponding facial area, (3) the absence of a posterior crossbite, (4) without mandibular deviation (the deviation of menton point less than 3 mm), (5) without a history of facial surgery or trauma, and (5) no systematic disease. The patients were divided into 2 groups according to the treatment plan. The patients who received nonextraction treatment were divided into the nonextraction group and those who received extraction of four premolars were divided into extraction group. The age and treatment duration were recorded from the mecidal history of the patients.

**Fig. 1:**
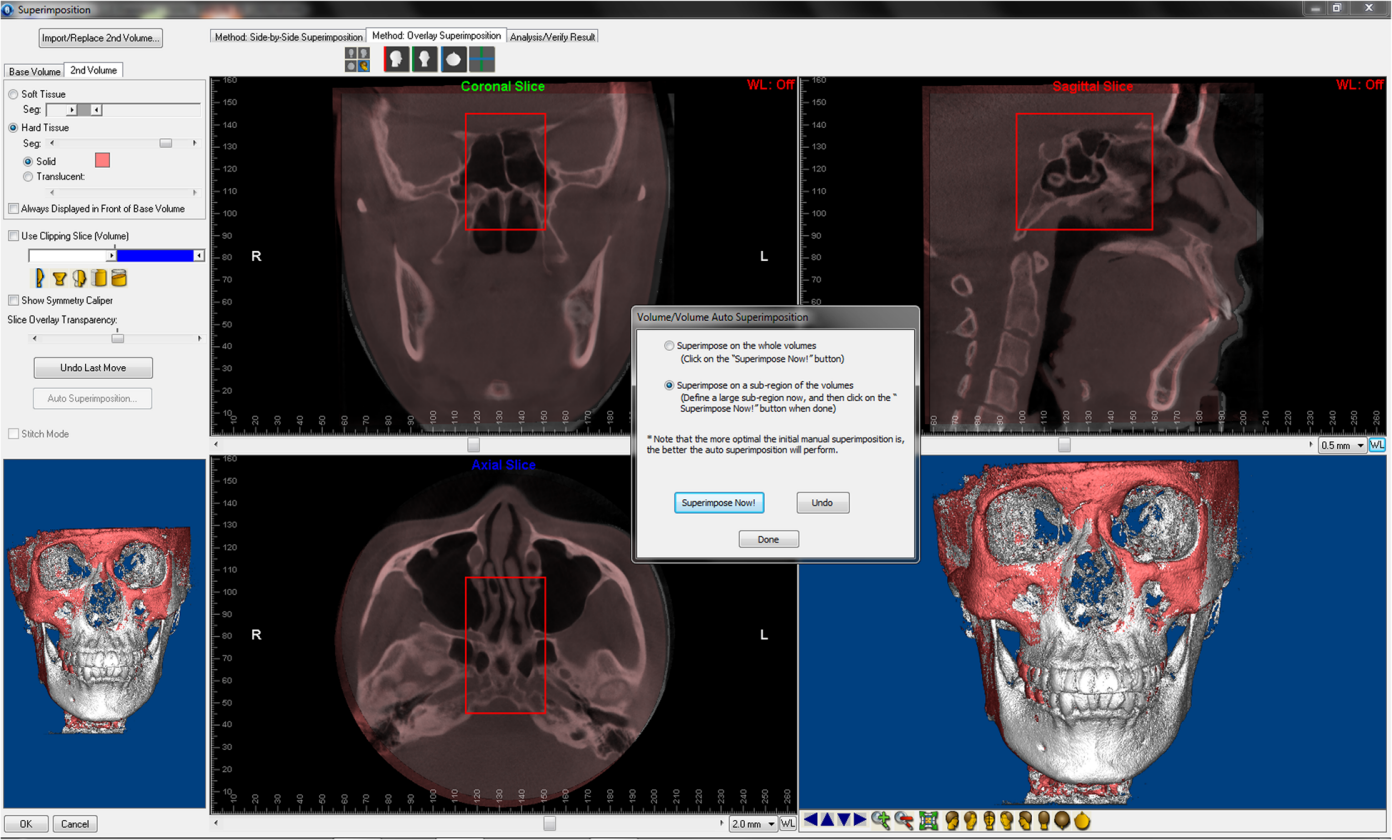
Superimposition of pre- and posttreatment CBCT scans based on the frontal base area (red subregion)

CBCT scans were taken by Newtom VGi (Quantitative Radiology, Verona, Italy) with the following settings: field of view, 24 × 19 cm; 90 kV; 6.0 mA; scan time, 15 s; and voxel size, 0.3 mm. Pretreatment and posttreatment CBCT scans were superimposed using Dolphin 11.8 Premium (Dolphin Imaging & Management Solutions, Chatsworth, CA, USA) [16] by frontal base area (Fig. 1). The reoriented posttreatment CBCT was exported in Digital Imaging and Communications in Medicine (DICOM) format. Pretreatment and reoriented posttreatment CBCT scans were used for segmentation, reconstruction and measurements.

The study was approved by the Institutional Review Board of Peking University School and Hospital of Stomatology (PKUSSIRB-201944062). Written informed consent was obtained from each patient before participation in the study.

### Cephalometric measurements

Cephalometric radiographs were reconstructed from pretreatment and reoriented posttreatment CBCT images. ANB and FMA (Frankfort-mandibular plane angle) were selected and measured to represent the sagittal and vertical skeletal relationship, respectively. The mandibular plane in FMA was defined as the tangent line to the lower margin of the mandible through the Menton point.

### Segmentation of the masseter muscle

**Fig. 2:**
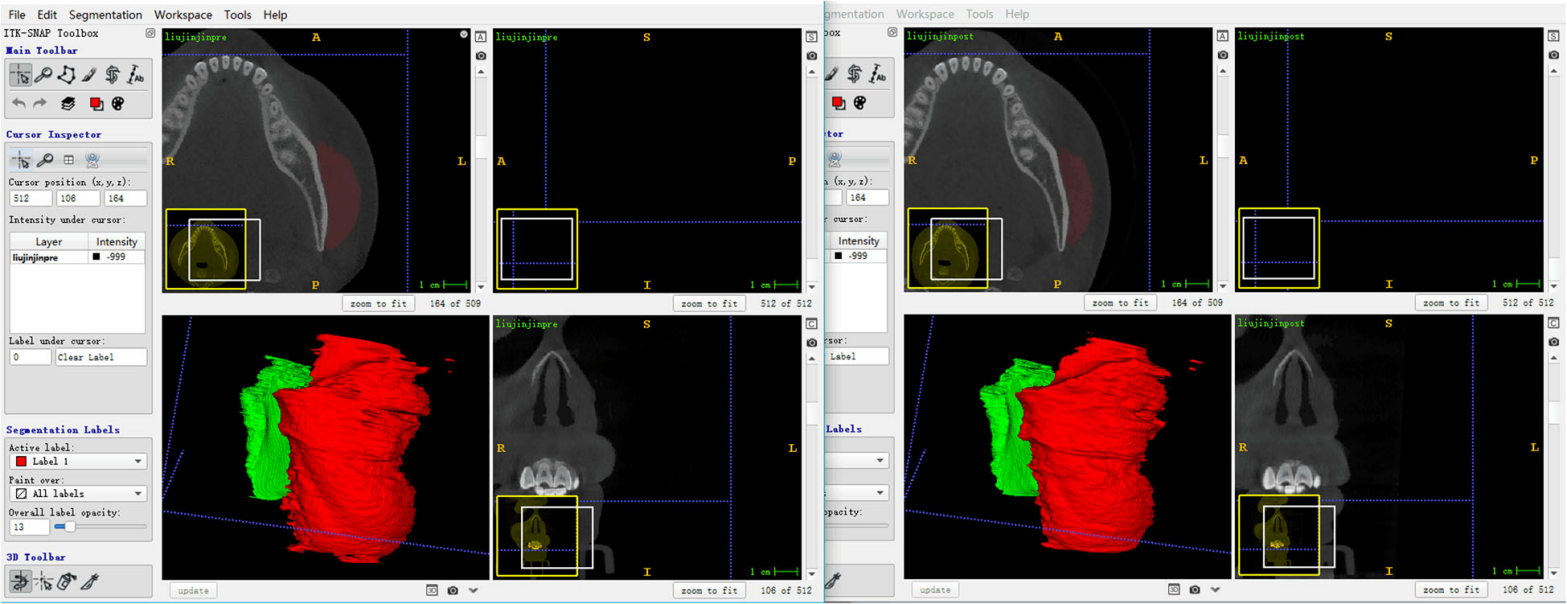
Pretreatment (left) and posttreatment (right) scans of one of the patients. Red: left masseter Green: right masseter

A self-developed generative adversarial network (GAN)-based framework [17] was used for noise reduction and automatic segmentation of masseter muscles from CBCT scans. The framework was developed by the Department of Machine Intelligence, Key Laboratory of Machine Perception (MOE), Peking University. To ensure the accuracy of segmentation, a layer by layer manual check was performed using ITK-SNAP 3.6.0 (http://www.itksnap.org) based on the automatic segmentation result. Pre- and posttreatment scans were placed in parallel and manually edited at the same time (Fig. 2) to ensure the consistency of anatomic structures. The left and right masseter muscle were separately exported and saved as a Stereo-Lithography Interface (STL) format.

### Reconstruction of the masseter muscle and facial 3D models

Threshold segmentation was used to generate facial surface 3D models. Pretreatment and reoriented posttreatment CBCT were imported into Mimics Research 22.0 (Materialise NV, Leuven, Belgium). The initial models of bilateral masseter muscles were imported and transferred to unify their own space coordinates with the systematic coordinates of the Mimics software. Then, the 3D models of the craniofacial bone structure, outer layer of the facial surface and the masseter muscle were calculated and saved as STL format.

### Measurement of the thickness of the facial soft tissues

**Fig. 3:**
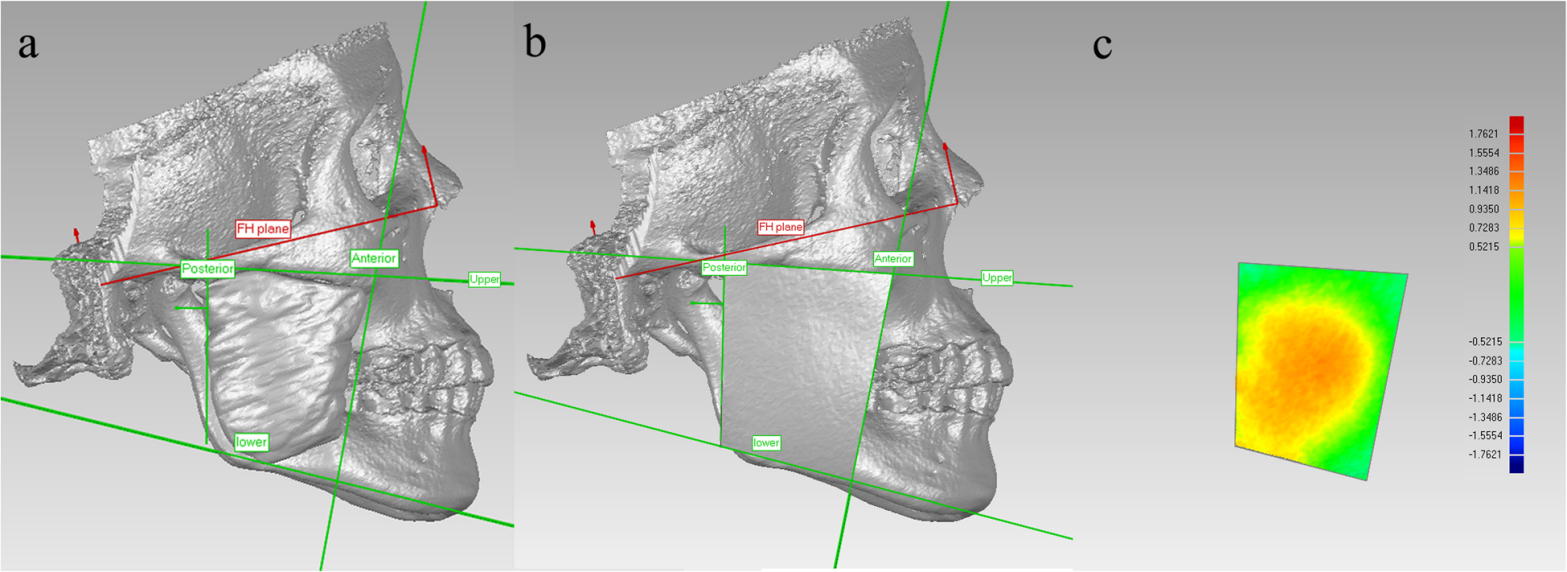
**a**. The boundary of the masseter area of the face (MAS); **b**. masseter area of the facial soft tissue (outer surface) (MAS); and **c**. deviation analysis between pretreatment and posttreatment MAS. Upper boundary: the tangent line passing the lower margin of the zygomatic arch; lower boundary: the tangent line passing the lower margin of the mandibular body; anterior boundary: the tangent line passing the anterior margin of the masseter muscle; posterior boundary: the line connecting the middle of the articular tubercle and the posterior point of the gonial angle

3D models of pre- and posttreatment craniofacial bones, facial surfaces and masseter muscles were imported into Geomagic Studio 14.0 (3D Systems Inc., Morrisville, NC, USA). The Frankfort horizontal plane (FH plane in Fig. 3a, b) was generated by the fitted plane of bilateral Orbitale points and Porion points. The sagittal plane was defined by the plane perpendicular to the FH plane and passing through the anterior nasal spine (ANS) point and posterior nasal spine (PNS) point. The boundaries of the masseter area of facial soft tissue (MAS) was defined using the reference lines as follows: upper boundary- the tangent line passing the lower margin of the zygomatic arch; lower boundary- the tangent line passing the lower margin of the mandibular body; anterior boundary- the tangent line passing the anterior margin of the masseter muscle; and posterior boundary- the line connecting the middle of the articular tubercle and the posterior point of the gonial angle (Fig. 3a). These lines were projected on the sagittal plane forming four reference planes vertical to the sagittal plane. The MAS was cut out from the facial model by the four reference planes (Fig. 3b). The average deviation calculated between the pre- and posttreatment MAS was used to represent the thickness change on each side (Fig. 3c). The mean value of the left and right sides was used to represent the change in thickness of MAS in one patient.

**Fig. 4:**
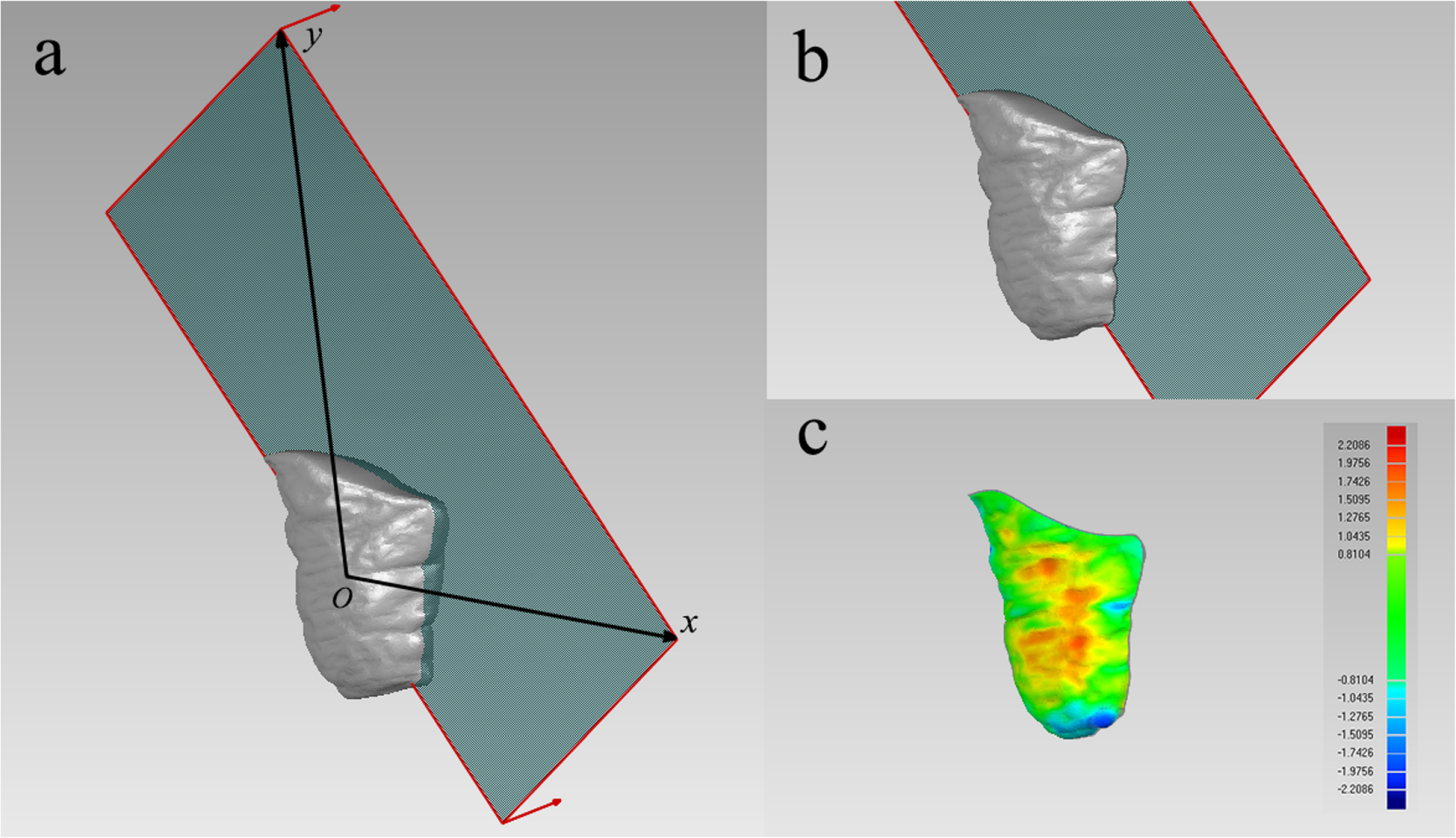
**a**. The anteroposterior principle component (x-axis) and superoinferior principle component (y-axis) of the MM; **b**. lateral half surface of the masseter muscle (MM); and **c**. deviation analysis of pre- and posttreatment MM

The masseter muscle was cut by a plane formed by its own superoinferior axis and anteroposterior axis (Fig. 4a) calculated by principal component analysis (PCA) and complied by MATLAB R2018b (MathWorks Inc., Natick, MA, USA) to ensure that only the lateral half surface of the masseter muscle was used for comparison (Fig. 4b). The average deviation calculated between the pre- and posttreatment lateral surface of masseter muscle was used to represent the thickness change on the left and right sides, respectively (Fig. 4c). The mean value of the left and right sides was used to stand for the thickness change of the masseter muscle in one patient.

Between the skin surface of the face and lateral surface of the masseter muscle lies the fat tissue (FT). In this study, we calculated total soft tissue (ST) thickness as the average deviation between the lateral surface of the masseter muscle and the corresponding facial surface. The change of FT thickness was calculated by subtracting the posttreatment ST thickness from the pretreatment ST thickness.

### Statistical analysis

The patients were divided into the extraction group and nonextraction group according to their treatment plan. The thickness changes of MAS, MM and FT were compared with zero. Pretreatment age, treatment duration, ANB, FMA and the thickness changes of facial soft tissues (MAS, MM and FT) were compared between the extraction and nonextraction groups using a t-test if the data were normally distributed; otherwise, the Mann-Whitney test was used. Pretreatment age, ANB angle and FMA were not in normal distribution, so Spearman’s correlation analysis was conducted among the abovementioned variables. In collinearity diagnostics, no significant multicollinearity was detected, so a linear regression model was used to confirm the correlations. In multiple linear regression analysis, extraction treatment, STpre, FMA, ANB, pretreatment age and treatment duration were listed as independent variables, among which, the extraction treatment was set as a dummy variable (extraction group = 1 and nonextraction group = 0) in the regression analysis. The FT, MM and MAS change were listed as dependent variables.

The sample size was calculated by Power Analysis and Sample Size (PASS) 15.0.1 software (NCSS LLC, UT, USA). To achieve a power of 0.80 in comparison between the two groups, at least 20 patients should be included in each group. And regression analysis required about 44 samples so that the power was about 0.81, therefore the two groups were analyzed as a whole (*n* = 42).

The intraclass correlation coefficient (ICC) was calculated to assess the reliability of the measurements. Since the calculation of the thickness change of the masseter muscle was conducted automatically by the computer program and software, which did not include random error, it was not repeated. The measurement of MAS, ANB and FMA was repeated once 2 weeks later by the same examiner and a second examiner, and the intra- and interexaminer ICCs were both larger than 0.99 for MAS measurement and between 0.98–0.99 for ANB and FMA measurement.

The statistical analysis was conducted using SPSS Statistics 23.0 (IBM Co., Armonk, NY, USA) at a significance level of 0.05.

## Results

### Descriptive statistics

In total, 42 patients were included in this study (extraction group: 22; nonextraction group: 20). The average age of the patients before the treatment was 25.62 ± 5.07 years. There was no significant difference in the average pretreatment age between the extraction group and the nonextraction group. The treatment duration was significantly longer in the extraction group than the nonextraction group. The cephalometric measurements of FMA and ANB are shown in Table 1 as well. To avoid the influence of FMA to the morphology of facial soft tissues, pre- and posttreatment FMAs were compared and no significant difference was found in both extraction group (*p* = 0.223) and nonextraction group (*p* = 0.135).

**Table 1:** Descriptive statistics in the extraction and nonextraction groups

Measurements	Groups		*p*-value
	Extraction	Nonextraction	
Treatment duration (d)	903.545 ± 170.390	679.909 ± 265.823	0.003**
Pretreatment age (y)	24.591 ± 4.043	27.227 ± 6.339	0.404
Pretreatment FMA (°)	25.091 ± 5.246	24.000 ± 5.561	0.517
Posttreatment FMA (°)	25.409 ± 5.561	24.355 ± 5.634	0.579
Pretreatment ANB angle (°)	3.759 ± 2.426	4.265 ± 3.374	0.577

*Abbreviations*: *d* Days, *y* Years

**p < 0.01

### Thickness of the masseter area of the face (MAS), masseter muscle (MM) and fat tissue (FT)

The change of MAS thickness is shown in Table 2. It shows that the thickness of the MAS significantly decreased during the treatment in both the extraction and nonextraction groups (*p* < 0.01). Compared to the nonextraction group, the thickness decrease of the MAS was significantly larger (> 1 mm) in the extraction group, which indicates that the facial morphology in the masseter area became more concave in the extraction group after treatment.

**Table 2:** Comparisons between the thickness changes (mm) of MAS, MM and FT

Measurements	Groups		*p*-value
	Extraction	Nonextraction	
Changes in MAS thickness (mm)	1.270 ± 0.780 (*p* < 0.001)	0.675 ± 0.822(*p* = 0.002)	0.021*
Changes in MM thickness (mm)	1.284 ± 0.586 (*p* < 0.001)	0.931 ± 0.658 (*p* < 0.001)	0.049*
Changes in FT thickness (mm)	0.006 ± 0.491 (*p* = 0.858)	− 0.302 ± 0.458 (*p* = 0.008)	0.096

*Abbreviations*: *MAS* Masseter area of the face, *MM* Masseter muscle, *FT* Fat tissue

*p < 0.05

This study showed that during treatment, the average thickness of the masseter muscle significantly decreased in both the extraction and nonextraction groups (*p* < 0.01) (Table 2). Similar to MAS thickness, the average thickness change of the masseter muscle was significantly greater in the extraction group than in the nonextraction group (*p* = 0.049).

No significant difference in the pretreatment ST thickness was found between the extraction (8.291 ± 1.291) and nonextraction group (8.406 ± 1.024). Additionally, its treatment change was not significantly different between the two groups (*p* = 0.096). In the extraction group, the change in the FT thickness was not significant from zero during treatment (Table 2, *p* = 0.858). However, in the nonextraction group, the FT thickness change was significantly smaller than zero during the treatment (Table 2, *p* = 0.008), which indicates that the FT thickness significantly increased during treatment.

### Correlation and regression analysis

Spearman’s correlation coefficients were calculated to initially evaluate the relationships between variables (Table 3). In the correlation analysis, a strong positive correlation was found between the thickness change of MAS and MM for the whole sample (*r* = 0.784, *p* < 0.01). A moderate positive correlation was found between the thickness change of MAS and FT (*r* = 0.533, *p* < 0.01). FMA was found to be significantly negatively related with the thickness change of both MM and MAS (*r* = − 0.309 and *r* = − 0.312 respectively, *p* < 0.05). STpre (pretreatment total soft tissue thickness) was positively correlated with FT change (*r* = 0.309, *p* < 0.05).

**Table 3:** Spearman’s correlation coefficients between treatment duration, pretreatment age, mandibular plane angle (FMA), ANB angle and thickness changes in MAS, MM and FT

	Duration	Age	FMA	ANB	MM	MAS	FT	STpre
Duration	/	−0.192	−0.115	− 0.038	0.010	− 0.066	−0.158	− 0.203
Age		/	−0.219	−0.005	0.108	0.090	−0.070	−0.065
FMA			/	0.275	−0.309*	−0.312*	− 0.013	0.236
ANB				/	−0.005	−0.198	− 0.276	−0.118
MM					/	0.784**	−0.031	− 0.059
MAS						/	0.533**	0.182
FT							/	0.308*
STpre								/

*p < 0.05

**p < 0.01

In multiple linear regression analysis (Table 4), extraction treatment and FMA both contribute significantly to MAS and MM loss during the treatment. STpre was significantly related to MAS change but its role was not significant in MM change.

**Table 4:** Results of the multiple linear regression analysis

	Included variables	Standardized coefficient (β)	Adjusted R^2^	*P* value
MAS	Extraction	0.435	0.325	< 0.01
	FMA	−0.438		< 0.01
	STpre	0.416		< 0.01
MM	FMA	−0.353	0.123	0.031
	Extraction	0.325		0.034
FT	Extraction	0.325	0.229	< 0.01
	Pretreatment age	0.301		0.049

**Fig. 5:**
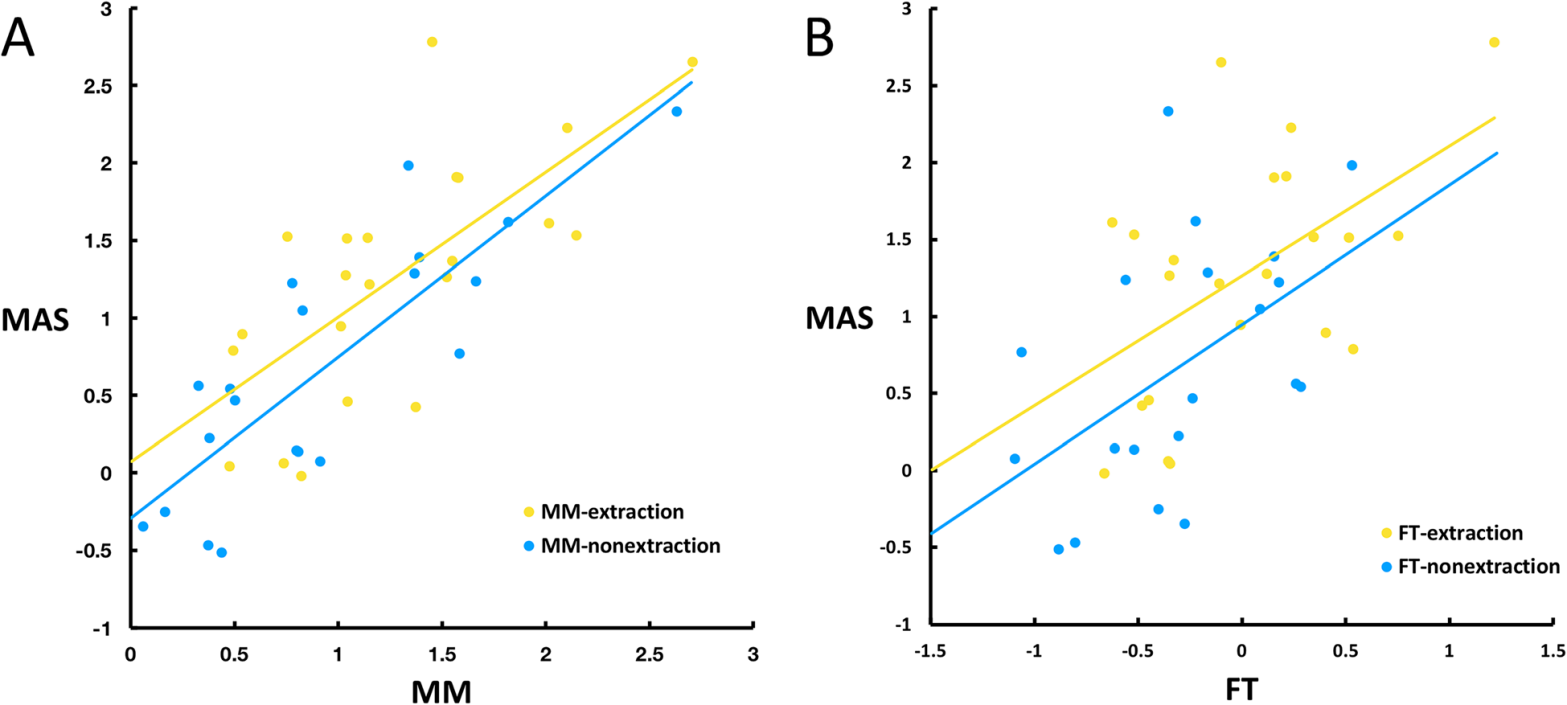
Scatter plots and regression lines between the thickness change of MAS and MM, FT.

The relationships between the dependent variables were revealed by scatter plots and regression lines (Fig. 5). MM and FT changes were both closely correlated with MAS change. The slope of the regression lines was larger in the non-extraction group than in the extraction group indicating that in the nonextraction group, MM and FT changes played a more important role in the MAS change.

## Discussion

For many years, orthodontists have been focusing on the improvement of the sagittal facial profile of patients during orthodontic treatments [1, 18]. However, patients also pay attention to changes in the appearance from the front during treatment. Previous results indicate that a decrease of the facial width [10], cheek flattening [9] and a reduction of 1–2 mm in the depth of the facial buccal area [5] tended to occur after orthodontic treatment, especially in extraction cases. Whether these changes are purely a result of fat loss or the morphological change of the muscle in this area, i.e., the masseter muscle, has not yet been investigated. In this study, we specifically explored the morphological change of the masseter area after orthodontic treatment. Pre- and posttreatment CBCT data were used to generate both a facial surface model and a masseter muscle model. As a result, the underlying mechanism for the ortho-related facial morphology change could be further studied.

In this study, hard tissue voxel-based CBCT superimposition was conducted to reveal the morphological change of MAS. The results showed that the thickness of MAS decreased significantly in both groups, and the decrease was greater in the extraction group than in the nonextraction group. Consistently, in other surface shape analysis [5, 9], a significant increase of concavity was also found in the cheeks in the extraction cases, whereas the changes in the nonextraction group were insignificant during orthodontic treatment. However, in the study by Dai et al. [5], the interested area was much closer to the nasolabial area, which could be largely influenced by changes in lip prominence. Ismail et al. [9] studied the overall facial surface shape in growing subjects (aged 11 to 19) in which growth could make a significant contribution to the overall change.

There are studies showing that occlusal force may change during orthodontic treatment [19, 20]. From this, we can infer that the morphology of the masseter muscles, which is one of the most superficial and massive muscles on the face, may influence the morphology of the face. In the past, magnetic resonance imaging (MRI) or computer tomography (CT) were commonly used to show the masseter muscle. MRI is more time-consuming and expensive, and metals, such as orthodontic appliances, may produce artifacts, restricting its use during orthodontic treatment. CT exposes the patients to a much larger amount of radiation, limiting its use in orthodontic area. CBCT has proven to be a better tool for 3D reconstruction with a lower radiation dose and high spatial resolution [21, 22]. The cross-sectional area of the masseter muscles was analyzed on CBCT scans by manual segmentation in cross sections in a study conducted by Lee et al. [15]. However, the soft tissue was less clear in CBCT images than in MRI or CT images, which makes it tedious work to manually segment the muscles from the CBCT. In this study, we used labels on CT images as training samples and applied machine learning to the autosegmentation of the masseter muscle to reduce the workload of manual segmentation, which greatly increases the efficiency and reliability [17]. We also compared the autosegmentation with the manual segmentation results and a relatively high similarity was found (mean DSC = 93.7%) [17], so the autosegmentation showed similar accuracy with manual segmentation. Since the internal surface of masseter muscle overlaps the temporal muscle and the internal pterygoid muscle [23] and origins from the mandibular ramus which is relatively irregular and stable in autosegmentation, we only compared the lateral surface of masseter muscle. Our study showed that the thickness of MM decreased significantly in both groups, and soft tissue loss was greater in the extraction group than in the nonextraction group. The changes in MAS and MM thickness during orthodontic treatment were strongly correlated to each other (*r* > 0.7). Significant differences were also found in the thickness change between the two groups. Both the thickness of the masseter muscle and the whole facial soft tissue showed a greater decrease in the extraction group than the nonextraction group, which is similar to the results of Dai et al. [5] who found that facial soft tissue thickness in the buccal region decreased more in the extraction group.

Many factors may influence the change of facial soft tissues during orthodontic treatment. Effects of sex and aging on facial soft tissues were observed in several studies [23, 24]. We included only female patients between 18 to 35 years old because this population seems to be more concerned with the influence of orthodontic treatment on their facial appearance. No significant correlation was found between the age or treatment duration with the soft tissue thickness change in this sample. Extraction treatment was extracted as a contributor to soft tissue loss during orthodontic treatment in the regression analysis. In addition, a moderate negative correlation was found between the FMA (mandibular plane angle) and the change in thickness of MM and MAS. It suggests that patients with a short face, usually recognized as having a larger masseter muscle and larger bite force [25–27], tend to lose more soft tissue during orthodontic treatment. In other words, the influence of the orthodontic treatment on facial morphology and possibly the bite force change may be larger in patients with a short face. In addition, the ANB angle (sagittal relationship) did not have much influence on soft tissue loss during the orthodontic treatment.

There are also limitations in this study. Firstly, though we only included the samples with body weight change less than 2 kg during the treatment, weight change still cannot be ruled out as an influencing factor of facial morphology change [11]. Secondly, the CBCT data of an untreated control group was lacked because the use of radiation examination on untreated patients may raise ethical issues. Therefore, normal change with time in the studied area would be combined with the treatment effect. A larger sample size and perspective design would be helpful for further extension of the results.

As to the esthetic effect, though statistically significant, an overall 1–2 mm average thickness change of facial soft tissues in the extraction cases and less than 1 mm thickness change in the nonextraction cases are of less clinical significance. In other words, this study verifies that orthodontic treatment won’t result in obvious soft tissue collapse in the cheek area in most of the female patients in their 20s to 30s. Nevertheless, there are still some patients whose soft tissue thickness decreased nearly 3 mm which may be noticed clinically. Therefore, patients with susceptible factors which were found in this study should be recognized and fully informed of the possible esthetic changes before treatment, especially when extraction was planned.

## Conclusions

There was a significant decrease in the thickness of the masseter area of the face (MAS) and the masseter muscles (MM) during orthodontic treatment. The decrease in the thickness of both the MAS and MM was larger in the extraction group than in the nonextraction group. The decreases in the thicknesses of MAS and MM were strongly correlated to each other, and the change in the thickness of MAS and FT were moderately correlated. Extraction treatment and small Frankfort-mandibular plane angle (FMA) significantly contributed to the thickness decreases of both MAS and MM and pretreatment thickness of fat tissue (FT) greatly contributed to MAS change during the orthodontic treatment.

## Data Availability

All data generated or analyzed during this study are included in this article. Any other information are available from the corresponding authors on reasonable request.

## Abbreviations

FMA: Frankfort-mandibular angle
MAS: Masseter area of the face
MM: Masseter muscles
FT: Fat tissue
STpre: Pretreatment total soft tissue thickness
d: Days
y: Years
